# Empirical examination of executive functioning, ADHD associated behaviors, and functional impairments in adults with persistent ADHD, remittent ADHD, and without ADHD

**DOI:** 10.1186/s12888-020-02542-y

**Published:** 2020-03-24

**Authors:** Belén Roselló, Carmen Berenguer, Inmaculada Baixauli, Álvaro Mira, Jose Martinez-Raga, Ana Miranda

**Affiliations:** 1 Developmental and Educational Psychology, University of Valencia, Valencia, Spain; 2 Catholic University of Valencia S. Vicente Martir, Valencia, Spain; 3 Unidad de Psiquiatría y Psicología Clínica, Hospital Universitario Dr. Peset, University of Valencia, Valencia, Spain

**Keywords:** Attention deficit hyperactivity disorder, Persistence, Remission, Functional impairments, Executive functioning

## Abstract

**Background:**

Previous studies suggest that childhood attention deficit hyperactivity disorder (ADHD) may continue in adulthood, producing adverse effects. Therefore, identifying factors that help to differentiate characteristics of ADHD persistence and remission has practical implications for evaluation and treatment. The first aim of this study was to analyze differences in executive functions (shift, working memory, inhibition, and plan/organize), symptoms associated with ADHD (inattention, hyperactivity, emotional lability, and self-concept), and functional impairments in adults with persistent ADHD (ADHD-P), with remittent ADHD (ADHD-R), and without ADHD (N-ADHD). The second aim was to study the contribution of functional impairments in these three groups based on executive functions and associated ADHD behaviors.

**Methods:**

Participants were 115 adults, 61 with a childhood ADHD diagnosis (40 persisters and 21 remitters) and 54 individuals with typical development. Self-reports were collected on executive functions, symptoms associated with ADHD, and functional impairments. Multivariate Analyses of Variance were conducted to test differences between the ADHD-P, ADHD-R, and N-ADHD groups on the evaluated variables. In addition, analyses were performed using two structural equation models with observed variables (path analyses).

**Results:**

The results indicated that significant executive and behavioral impairments and adverse functional outcomes in different life domains are related to the diagnostic persistence of ADHD. Recovery from the disorder is associated with better results, although hyperactivity/restlessness behaviors and plan/organize deficits continue to be present in remitter individuals.

**Conclusions:**

The ADHD-P and ADHD-R groups showed some differences in their executive, behavioral, and functional impairments. Furthermore, the impairments in each group can be predicted by different executive functions and other symptoms associated with the disorder. These results should be taken into account in order to improve clinical practice.

## Background

ADHD is a disorder that frequently continues in adulthood. For many people, it is chronic, even though the data on its persistence from follow-up studies are not congruent, with percentages ranging from 4 to 77%. This great variability may be due, above all, to the variety of methods and cut-off points established to diagnose ADHD, the assessment procedures used, the age when beginning the study and follow-up, and the requirement (or not) of the presence of impairments. A recent systematic review concluded that, although few persistence studies have applied strict criteria, those that have done so found ADHD persistence rates close to 50% [1], which could increase to 86.5% in the combined presentation [2].

Adults with ADHD suffer from a pattern of functional impairment in a wide variety of daily work and academic activities, multiple risky behaviors [3], financial problems, dependence on public aid, and risk of poverty [4]. The social and personal importance of these data has led to the interest in studying the trajectory of the disorder and factors associated with its persistence or remission. However, the complexity of the manifestations of ADHD and the diversity of the outcomes are not explained by a simple impairment, and a range of cognitive mechanisms have been proposed, including [5]: aversion to delay, dysfunctional responses to contingencies, increased intraindividual variability in response time due to attentional fluctuations or overall slow cognitive processing speed, and executive functioning impairments (EF).

The executive system includes a broad range of processes associated with the prefrontal and thalamic-reticular areas of the brain, responsible for directing and regulating cognitions, emotions, and behavior in order to reach a desired goal. There is evidence that EF deficits may be core components of the complex neuropsychology of ADHD [6]. Support for this proposal can be found in the EF impairments that have been identified in adults with ADHD on both neuropsychological tasks [7] and standardized behavior rating scales [3]. Much less clear is the influence of EF on the persistence/remission of ADHD. An extensive review of studies that used neuropsychological tests [8] concluded that ADHD-P and ADHD-R groups in adulthood continued to show differences in their performance, compared to controls, with the effect being less severe in ADHD-R. In addition, ADHD-P and ADHD-R did not differ on higher level neurocognitive functions that require more consciously controlled processing, or on lower level neurocognitive functions linked to more automatic processing and the need for less mental effort.

An analysis focusing on the role of the executive processes that have more discriminatory power [9] in the persistence/remission of the ADHD reveals that not all the studies [10, 11], but the majority, found that the ADHD-P group shows significantly worse performance than the ADHD-R group on neuropsychological tests of inhibition [12, 13], working memory [14], attention-vigilance [15], or set shifting [16]. The standardized behavior rating scales have yielded a consistent view, highlighting that the ADHD-P group shows more severe deficits than both the ADHD-R and community groups on ratings of time management, self-organization, inhibition, self-motivation, and self-activation. In addition, most ADHD–P cases fell in the clinically impaired range on self-reported EF in comparison with a minority of ADHD–R. Nevertheless, the ADHD-R group had significantly worse scores than the community group (without ADHD) on self-organization, inhibition, and self-activation [17]. Likewise, EF measures are linked to symptom persistence [18], and they are good predictors of impairments in major life activities and occupational functioning in adult studies of ADHD [17].

On the other hand, without a doubt, adults with ADHD commonly have additional problems, such as feelings of restlessness, unfocused mental activity and memory problems, emotional dysregulation, or low self-esteem [19–22]. These problems could be linked to the persistence of the disorder, adversities in everyday functioning [23, 24], and poor quality of life [25]. The ADHD-P group, in comparison with ADHD-R, reports more emotional impulsivity, which contributes to impairments in occupational, educational, criminal, and financial outcomes beyond the ADHD symptoms [26]. ADHD-P are more likely than ADHD-R to have comorbidity, as well as functional impairments in academic, emotional, and interpersonal domains [27]. The persistence of inattention /memory problems, such as losing or forgetting necessary things, difficulties in paying attention when necessary, or simply listening, has a negative effect on important areas of daily functioning in adults with ADHD [28]. Likewise, ADHD-P group have worse functioning and elevated rates of diverse comorbidities compared to ADHD-R group. However, although to a lesser degree, remitter individuals remain impaired in certain domains, and they experience impairments at home and with friends [29].

In summary, in the past decade, evidence has been accumulated about the differences between adults with a childhood ADHD diagnosis and N-ADHD in EF [3, 7] and problems related to inattention and restlessness, self-concept, and impulsivity/ emotional lability [20–23]. However, less is known about the magnitude and nature of the functional impairments of ADHD-P and ADHD-R groups or the role played by the EF and other behaviors linked to the disorder. The current study aims to characterize the cognitive and behavioral manifestations of adults with ADHD-P and ADHD-R by using standardized self-report measures because they are sensitive to detecting executive problems in unstructured environments [17], and they are strongly associated with impairments in major life activities [30].

The specific aims were: (1) to analyze differences in EF (inhibition, shift, working memory, and plan/organize), behaviors associated with ADHD (inattention/memory. Hyperactivity/restlessness, emotional lability, and self-concept), and general functional impairments in ADHD-P, ADHD-R, and N-ADHD adults; and (2) to examine the contribution of EF and ADHD associated behaviors to the functional impairments of adults with ADHD-P and ADHD-R. We expected that the ADHD-P group would exhibit greater EF deficits and severity of behavioral symptoms linked to the disorder than the ADHD-R and N-ADHD groups. On the other hand, the ADHD-R group would present better EF and fewer ADHD associated behaviors than the ADHD-P, although they would present more deficits than the N-ADHD group. We also expected that the EF and behaviors associated with ADHD would contribute to the general functional impairment of the two groups with ADHD and the N-ADHD group.

## Methods

### Study design and procedure

This is a descriptive cross-sectional design between a group of adults with a childhood ADHD diagnosis twelve years after the initial diagnosis and a N-ADHD group, that were matched on sex, age and IQ estimated Participants with ADHD came from the Spanish sample from the International Multicenter ADHD Genetics (IMAGE) study, a prospective study of children with ADHD with combined subtype (ADHD-C), recruited between 2003 and 2006. Clinical diagnosis was based on combining information from the Parental Account of Childhood Symptoms [31] interview and the DSM-IV items on Conners’ parent and teacher questionnaires [32]. Exclusion criteria included a diagnosis of autism, epilepsy, IQ < 70, or brain disorders [33].

On average, 12 years later, from 2013 to 2017, contact was again made with the families to request their collaboration. Youths with ADHD and a family informant were given verbal and written information about the study to be carried out. Sixty-one individuals gave their consent to participate before beginning the first evaluation session in the present follow-up phase (Mean age at baseline =9.4 ± 2.3, and Mean IQ = 106 ± 16.9). The retention rate was 75.31% (61 from 81): thirteen subjects (16.05%) did not participate because we could not locate them, and seven declined the invitation to attend the evaluations. There were no differences in ADHD severity between the 61 children who continued in the follow-up and the 20 who did not participate, based on the parents’ (*t* (79) = − 0.60, *p* = .549) or teachers’ (*t* (79) = 0.37, *p* = .712) ratings on the DSM-total, the gender (χ^2^ = .203; *p =* .652*)* or educational level of the parents (t = (79) = −.970, *p* = .334).

Data for the present study were gathered in two evaluation sessions held in an office that met optimal conditions in the Faculty of Psychology at the University. A clinical psychologist with accredited experience and a senior investigator performed the assessment in separate sessions with the participant and a family informant. The participants were given the instructions as they appear in the respective manuals of questionnaires, and they filled them out in approximately 50-min sessions. Everyone who was taking medication as part of the diagnosis stopped taking it 48 h before the evaluation and on the two days it lasted.

### Sample characteristics

Sixty-one adults with ADHD and 54 controls participated in the present study. Adults with ADHD had ages ranging from 18 to 24 years (M = 18.7; SD =1.3), with IQs within the average range (M = 105.2; SD = 13.8). Of them, 95.1% were men, and 4.9% were women. Most of the subjects (> 80%) had taken medication, mainly stimulants, at some point in time.

ADHD symptoms were measured with the Conners Adult Rating Scale [34] (CAARS), using the DSM-IV inattention and hyperactivity/ impulsivity subscales of the observer and self-report versions. Presence of an ADHD symptom was determined by a score of two or more on the four-point CAARS scales (0 = never, 1 = once in a while, 2 = often, 3 = very frequently) when endorsed by either a parent or the participant. Persistence of ADHD in adulthood was defined using the DSM-5 [35] symptom cut-off criteria (at least five inattentive or five hyperactive-impulsive symptoms). Forty subjects (65.6%) were identified as ADHD-P, whereas 21 (34.4%) were classified as ADHD-R because they met the ADHD-C criteria at baseline, but not at follow-up. Remission was higher than the 14.8% found in another previous study carried out in pre-adolescence [36]. Our data support the “diagnostic stability” of ADHD, but with a tendency to decline with age [37].

The N-ADHD group was composed of 54 young adults who were selected according to the criteria of the Spanish National Institute of Statistics for the distribution of the population between 18 and 24 years old, based on their grade level and employment status. We contacted them by spreading information about this research project in universities, vocational training schools, and secondary schools, and participation was voluntary. All the individuals met the following criteria: a) no history of significant inattention or hyperactivity-impulsivity problems; b) T scores below 65 on Conners’ ADHD subscales; c) absence of neurological disorders, sensory or motor impairments, autism, or psychosis; d) IQ equal to or greater than 70.

The three groups, ADHD-P, ADHD-R, and N-ADHD, were matched on sex χ^2^ = 2.81, *p* = 0.24), age (F_(2,112)_ = 2.85, *p* = 0.07), and estimated IQ (F _(2,112)_ = 1.62, *p* = 0.20).

### Measures

#### ADHD rating behavior

The self-rating form of the CAARS Long Version (CAARS) [33] used in this investigation consists of 66 statements rated on a scale from 0 to 3, where 0 = *not at all, never,* and 3 = *very much, very frequently*. Three of the eight subscales correspond exactly to the *DSM*-*IV* diagnostic criteria for ADHD and its subtypes [38]: *DSM-IV* inattention; *DSM-IV* hyperactivity-impulsivity; and *DSM-IV* Total. The other five scales measure behaviors that have been found to be associated with ADHD: inattention; hyperactivity; impulsivity/emotional lability, problems with self-concept, and Conners’ ADHD Index.

Test–retest reliability has been found to be acceptable, and it has been shown to be valid in distinguishing individuals with ADHD from healthy controls [39]. In the present study, we used the scores on the inattentive, hyperactive, impulsivity/emotional lability, and problems with self-concept scales, and α coefficients for the individual subscales in our sample ranged from .71 for DSM hyperactivity/impulsivity to .92 for impulsivity/ emotional lability.

#### Executive functions

The Behavior Rating Inventory of Executive Functions–Adult version [40], BRIEF-A, was used to obtain self-reports of cognitive, behavioral, and emotional EF from the participants in everyday situations. Items are rated on a three-point scale (never, sometimes, often), with higher scores indicating greater EF impairment in daily life. It includes nine factorial subscales: inhibit, shift, emotional control, self-monitor, initiate, working memory, planning/organize, task monitor, and organization of materials. Higher scores on each scale indicate greater EF impairments. The nine subscales form two separate indexes, the behavioral regulation index (BRI) and the metacognition index (MI).

In this study, the inhibit and shift subscales from the BRI were selected, as well as the working memory and plan/organize subscales from the MI, which are good predictors of ADHD status [9]. The inhibit scale measures the ability to inhibit, resist, or not act on an impulse. The shift scale measures the ability to make transitions, problem solve flexibly, switch attention, and change the focus from one mind set or topic to another. The working memory scale assesses the capacity to actively hold information in the mind for the purpose of completing a task or generating a response. The plan/organize scale reports the ability to manage current and future-oriented task demands within the situational context [40].

The BRIEF-A has adequate test–retest reliability (correlations ranging from .82 to .94) and internal consistency (α coefficients ranging from .85 to .98), as well as convergent and discriminant validity [40]. In this study, α coefficients for the subscales used ranged from .77 to .88.

#### Functional impairment

The participants filled out the Weiss Functional Impairment Rating Scale Self-report, WFIRS-S [41], which is considered an appropriate self-report instrument for adolescents and adults. The scale includes 69 items rated on a four-point Likert-type scale with responses ranging from *never or not at all* to *very often or very much*. The items cover the subdomains of family (“Causing fighting in the family”), life skills (“Problems managing money”), self-concept (“Feeling frustrated with yourself”), social activities (“Trouble getting along with people”), and risky activities (“Breaking or damaging things”). Total impairment is indicated by the sum of the subdomains with impairment (e.g. two items scored as often/a lot or one scored as quite often/very much per subscale). In our study, the work subscale was not included due to the low number of subjects who were working at the time of the evaluation. Self-concept was not taken into account either, in order to avoid overlap with items on the CAARS subscale.

Psychometric properties of the WFIRS-S, including construct validity, internal structural validity, and external validity, have been tested in several studies [42, 43]. Cronbach’s alpha coefficients obtained for the overall scale have been high (.91). In the present research, the α coefficient was .85 for the overall scale (between risky activities = .81 and life skills = .70).

The Vocabulary and Block Design subtests from the Wechsler Adult Intelligence Scale [44] were administered to evaluate the cognitive ability of all the participants.

### Data analyses

Normality and Q-Q graphics screening was carried out, with the distance from normality not requiring the application of non-parametric tests. Multivariate Analyses of Variance (MANOVAs) were conducted to test differences between the ADHD-P, ADHD-R, and N-ADHD groups in EF, characteristics related to ADHD, and functional impairments. Significant group effects from the MANOVAs were followed up with post hoc group comparisons using the Tukey test HSD. Bonferroni correction was also applied.to control type I error due to multiple comparisons. The proportion of total variance accounted for by the independent variables was calculated using partial eta squared (eta squared, .05 = small; .06–.14 = medium; .14 = large) [45].

In addition, analyses based on the specification of two structural equation models with observed variables (path analyses) were carried out. The first model explores total functional impairment with four predictors: Inhibition, shift, working memory, and planning. The second model explores total functional impairment with four other potential predictors: Inattention/memory, hyperactivity, impulsivity/emotional lability, and problems of self-concept. The two models have been specified and tested in Mplus 8.3 [46], with Robust Maximum Likelihood as the estimation method. Model fit was evaluated with the chi-square statistic, the Comparative Fit Index (CFI), and an index based on residuals, the Square Root Mean Residual (SRMR). The following cut-off criteria were used to determine good fit: CFI above .90 (better when higher than .95), SRMR below .08 [47].

After establishing a proper model for each group, a multi-group routine was implemented, with the purpose of testing for moderation effects due to group (ADHD-P, ADHD-R, and N-ADHD). In a first step, a baseline model is tested simultaneously in the three samples, with no constraints across samples, that establishes the best fit for the multi-group routine. Then, a second multi-group model with all the effects constrained across all groups is tested, and it is first compared to the baseline model. The models can be compared using two rationales: tests for chi-square differences between the different models and practical fit usually determined with CFI differences of less than .01 or .05 indicating that the models have the same fit [48].

## Results

Differences between the ADHD-P, ADHD-R, and N-ADHD groups on executive functions, ADHD associated behaviors, and functional impairments.

**Fig. 1:**
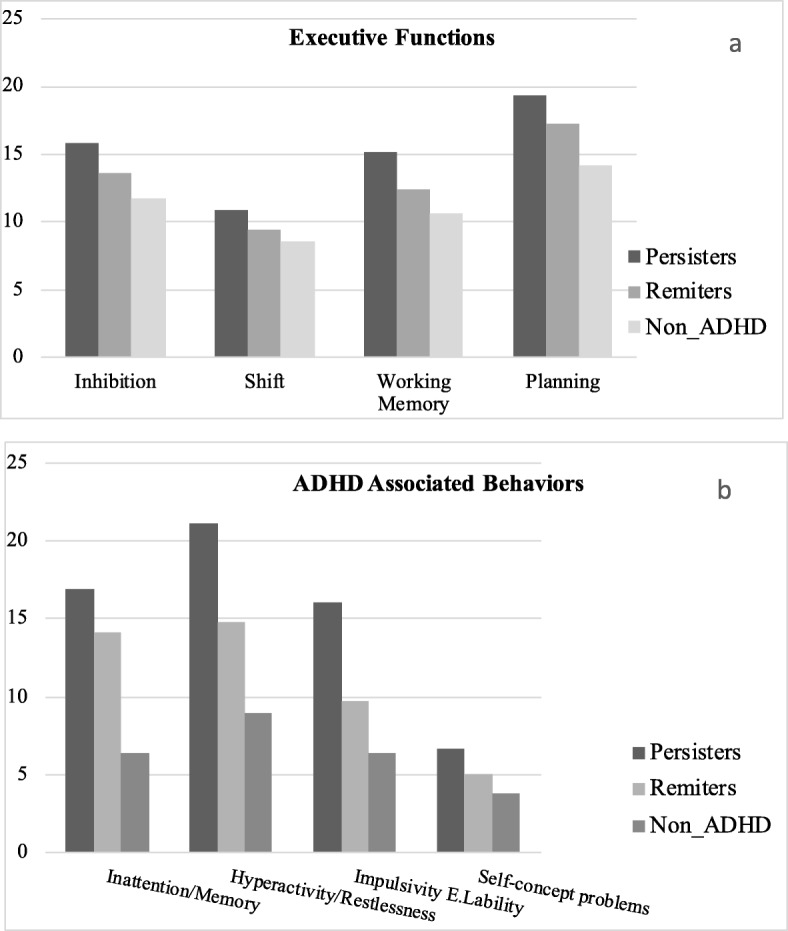
EF and associated behaviors. Mean scores in EF (1**a**) and ADHD associated behaviors (1**b**) in ADHD-P, ADHD-R, and N-ADHD groups

The MANOVA performed to evaluate the differences in EF between the ADHD-P, ADHD-R, and N-ADHD groups was statistically significant [Wilk‘s Lambda (Λ) = .57, 293 F(2,112) = 8.69, *p* < .001, η2 *p* = .24]. Post hoc group comparisons showed statistically significant values for inhibition, shift, working memory, and plan/organize (see Table 1 and Fig. 1). On inhibition and shift, the ADHD-P group obtained significantly higher scores than the N-ADHD group, which indicated greater problems. The ADHD-P group also scored on working memory higher than the other two groups, ADHD-R and N-ADHD, Finally, the ADHD-P group and the ADHD-R group had significantly higher scores on plan/organize than the N-ADHD group, with both ADHD groups showing more problems on this executive function.

**Table 1:** Mean (M), standard deviation (SD), and ANOVAs between the ADHD-P, ADHD-R, and N-ADHD groups on executive functions and ADHD associated behaviors

	1.ADHD-P *N* = 40	2.ADHD-R *N* = 21	3.N-ADHD *N* = 54			Group Difference
	M (SD)	M (SD)	M (SD)	F_(2,112)_	η^2^p	
Executive Functions						
Inhibition	15.85 (2.87)	13.62 (2.88)	11.72 (2.63)	25.55**	.31	1 > 3
Shift	10.85 (3.11)	9.43 (5.59)	8.56 (2.23)	9.07**	.13	1 > 3
Working Memory	15.20 (3.70)	12.43 (2.97)	10.69 (2.09)	27.84**	.33	1 > 2, 3
Plan/Organize	19.38 (4.99)	17.24 (3.66)	14.13 (2.99)	21.10**	.27	1 > 3; 2 > 3
ADHD Associated Behaviors						
Inattention/Memory	16.93 (7.01)	14.14 (8.78)	6.36 (4.28)	32.90**	.37	1, 2 > 3
Hyperactivity/Restlessness	21.15 (5.80)	14.81 (5.59)	8.91 (4.83)	60.82**	.52	1 > 2, 3; 2 > 3
Impulsivity/ E. Lability	16.08 (6.84)	9.76 (7.46)	6.33 (5.16)	28.17**	.33	1 > 2, 3
Self-concept problems	6.63 (5.31)	5.00 (3.97)	3.78 (3.55)	4.99*	.08	1 > 3

Bonferroni correction, p* < .05, p** < .01

The higher scores on EF indicate greater EF impairments

The MANOVA performed to evaluate the differences in ADHD associated behaviors among the three groups was statistically significant [Wilk‘s Lambda (Λ) = .40, F_(2,112)_ = 15.82, *p* < .001, η2 *p* = .36]. Post-hoc analysis showed statistically significant differences in inattention/memory, hyperactivity/restlessness, impulsivity/emotional lability, and self-concept problems. The two ADHD-P and ADHD-R groups presented inattention/memory problems that were statistically higher than the N-ADHD group. The ADHD-P group presented greater problems than the other two groups, ADHD-R and N-ADHD, on hyperactivity/restlessness and impulsivity/emotional lability, and it obtained significantly higher scores than the ADHD-R group on self-concept problems. However, the ADHD-R group was only statistically different from the N-ADHD group on hyperactivity/restlessness.

**Fig. 2:**
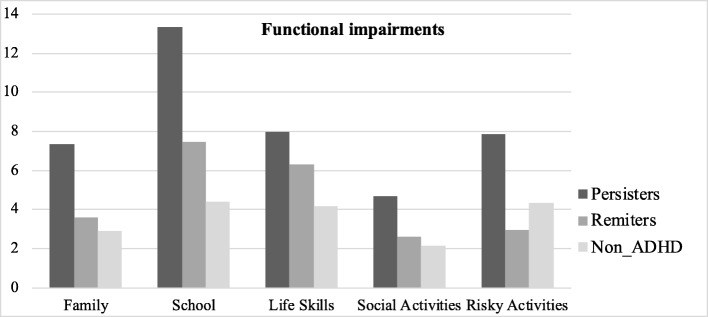
Functional impairments. Mean scores in functional impairments in ADHD-P, ADHD-R, and N-ADHD groups

Finally, the MANOVAS carried out on the functional impairment domains also indicated the existence of significant differences [Wilk‘s Lambda (Λ) = .50, F_(2,112)_ = 7.38, *p* < .001, η2 *p* = .29]. According to the results of the post-hoc analyses (Table 2 and Fig. 2), the differences between groups crossed all the domains: family, daily life skills, social activities, risky activities, and total impairment. The ADHD-P group presented more functional problems than the ADHD-R and N-ADHD groups in the domains of family, school, risky activities, and total impairments. Furthermore, the impairments in life skills and social activities in the ADHD-P group were greater than those of the N-ADHD group.

**Table 2:** Mean (M), standard deviation (SD), and ANOVAs between the ADHD-P, ADHD-R, and N-ADHD groups in functional impairments

	1.ADHD-P *N* = 40	ADHD-R *N* = 21	3.N-ADHD *N* = 54			Group Difference
	M (SD)	M (SD)	M (SD)	F_(2,112)_	η^2^p	
Family	7.35 (4.48)	3.61 (2.63)	2.87 (2.43)	21.90**	.28	1 > 2, 3
School	13.32 (7.90)	7.47 (5.01)	4.42 (3.65)	28.07**	.33	1 > 2, 3
Life Skills	7.95 (5.24)	6.28 (4.42)	4.18 (3.53)	8.68**	.13	1 > 3
Social Activities	4.67 (3.67)	2.62 (2.95)	2.14 (2.35)	8.61**	.13	1 > 3
Risky Activities	7.87 (5.85)	2.95 (2.99)	4.33 (3.85)	10.49**	.15	1 > 2, 3
TOTAL	44.37 (21.45)	26.04 (16.19)	19.92 (13.00)	24.46**	.30	1 > 2, 3

Bonferroni correction p** < .01

Analyzing functional impairment with EF of Inhibition, shift, working memory, and plan/organize.

First, a path analysis was estimated to explore functional impairment in the whole sample, in order to establish a model that fit the data. This model fit the data very well: χ^2^(1) = .761, *p* = .38, CFI = 1, SRMR = .008.

The multi-group routine starts by estimating the model simultaneously in the three groups (ADHD-P, ADHD-R, N-ADHD), but without constraints, where all the parameters in each group are free. This model adequately fit the data: χ^2^(3) = .796, *p* = .85, CFI = 1, SRMR = .01. Then, all the effects of the predictors on total functional impairment were constrained to be equal to those of the N-ADHD group. That is, the four effects of the predictors were estimated in the N-ADHD group and then fixed to those values in the other groups. Model fit deteriorated: χ^2^(9) = 6.66, *p* = .67, CFI = .98, SRMR = .049. Therefore, a sequence of models was estimated in which one parameter at a time was unconstrained until a model with the same (or better) fit as the baseline model (unconstrained) was found. The best fitting model showed the moderation of the effects of working memory on total functional impairment (χ^2^(7) = 2.47, *p* = .92, CFI = 1, SRMR = .02.

In this model, the effects of inhibition, shift, and working memory on functional impairment were equal across groups. The effects of shift and working memory were not statistically significant: β = .132, *p* > .05 and β = .103, p > .05, respectively. The effect of inhibition was positive and statistically significant (β = .289, *p* < .001). The effect of plan/organize on functional impairment was group-specific (a moderation effect of group). Specifically, the effect of this function on impairment was .013 (p > .05) in the N-ADHD group, .292 (*p* = .012) in the ADHD-R group, and .357 (p < .001) in the ADHD-P group. In sum, plan/organize affected the total impairment more in the ADHD-P group than in the ADHD-R group, and this effect was not statistically significant in the N-ADHD group. R-squares in the three groups (N-ADHD, ADHD-R, ADHD-P) were, respectively: .18, .34, and .28.

Analyzing functional impairment with ADHD behaviors of inattention, hyperactivity/ restlessness, impulsivity/emotional lability, and self-concept problems.

A second path analysis to examine functional impairment using new predictors (inattention, hyperactivity, impulsivity/emotional/lability, and self-concept) was estimated in the whole sample, in order to establish a model that fit the data. This model fit the data well: χ^2^(1) = 9.55, *p* = .002, CFI = .91, SRMR = .087.

The multi-group routine starts with the model estimated simultaneously in the three groups (ADHD-P, ADHD-R, N-ADHD), but without constraints, where all the parameters in each group are free. This model adequately fit the data: χ^2^(3) = 10.11, *p* = .017, CFI = .92, SRMR = .10. Then, all the effects of the predictors on functional impairment were constrained to be equal to those in the N-ADHD group. That is, the four effects of the predictors were estimated in the N-ADHD group and then fixed to those values in the other groups. Model fit considerably deteriorated: χ^2^(9) = 28.34, *p* < .001, CFI = .76, SRMR = .13. Accordingly, a sequence of models was estimated in which one parameter at a time was unconstrained until a model with the same (or better) fit as the baseline model (unconstrained) was found. The model with the best fit showed the moderation of the effects of inattention and impulsivity/lability on total functional impairment (χ^2^(7) = 6.35, *p* = .49, CFI = 1, SRMR = .046.

In this model, the effects of hyperactivity/restlessness and self-concept on impairment were equal across groups. The effect of hyperactivity/restlessness was not statistically significant (β = .095, *p* > .05), whereas the effect of self-concept was positive and statistically significant (β = .236, *p* = .003). However, the effects of inattention and impulsivity/emotional lability on functional impairment were group-specific (moderation effect of group). Specifically, the effect of inattention on impairment was .292 (*p* = .009) in the N-ADHD group, −.108 (p > .05) in the ADHD-R group, and .384 (*p* < .001) in the ADHD-P group. In sum, inattention affected functional impairment more in the ADHD-P group than in the N-ADHD group, and this effect was not statistically significant in the ADHD-R group. Regarding the effect of impulsivity/emotional lability on total impairment, the effect was .286 (*p* = .039) in the N-ADHD group, .611 (p < .001) in the ADHD-R group, and .225 (p < .001) in the ADHD-P group. In summary, the effect of impulsivity/emotional lability was much larger in the ADHD-R group than in the other two groups, which did not significantly differ on this effect. R-squares in the three groups (N-ADHD, ADHD-R, and ADHD-P) were .43, .55, and .41, respectively.

## Discussion

The current study aimed to extend the understanding of the persistence and remission of ADHD by integrating multi-level information through the evaluation of executive processes, behavior problems, and functional impairments in different domains (family, school, life skills, social activities, and risky activities). A first objective, comparing ADHD-P, ADHD-R, and N-ADHD, was to identify which executive and behavioral problems are present in adults with persistent and remittent ADHD and determine whether recovery is associated with a better development. It was hypothesized that ADHD-P individuals would display more executive impairments, more severe problems commonly associated with the disorder, and more functional impairments than the ADHD-R and N-ADHD groups. It was also hypothesized that ADHD-R individuals would present better functioning in different areas than ADHD-P, although they would display some deficiencies compared to the N-ADHD group.

As expected, our results indicated that the ADHD-P group, in comparison with the N-ADHD group, showed differences on all the executive functions measured by self-reported EF ratings: inhibition, shift, working memory, and plan/organize, coinciding with findings from another previous study17]. By contrast, the ADHD-R group only obtained significantly worse EF mean scores than the N-ADHD group on the plan/organize scale, which evaluates aspects related to self-organization and planning ahead for future activities. The presence of these kinds of deficits highlights the stability that characterizes general problems in individuals with a childhood diagnosis of ADHD related to work organization, prioritizing activities, or planning activities. There were no significant differences between ADHD-P and ADHD-R on inhibition, shift, or plan/organize, although when comparing the two ADHD groups, a worse level was found on these functions in the ADHD-P group.

Another important point has to do with the presence of the working memory problems in adults with ADHD, which is consistent with the image provided by a complete meta-analytic review of experimental studies [49]. The working memory impairment was not noted at the same level in the two groups with ADHD. In fact, as mentioned above, it was the only one of the four executive functions on which the ADHD-P group performed worse than the ADHD-R group, suggesting that it could be a relevant factor in the persistence of the disorder in adulthood. In any case, the working memory impairment cannot be considered a specific characteristic in ADHD. It has been noted in a broad spectrum of psychopathologies and, specifically, constitutes a cross-sectional deficit that ADHD shares with other neurodevelopmental disorders, such as autism, learning disabilities, or specific language disorders [50].

Regarding behaviors associated with ADHD, as expected, ADHD-P individuals, compared to N-ADHD individuals, also showed greater severity in the behaviors of inattention, hyperactivity, emotional lability, and self-concept problems. Moreover, impaired emotional regulation, which has been proposed as an essential component of ADHD [51], also showed differences between the two groups with ADHD. The persister individuals reported a greater tendency to feel frustrated when facing small difficulties, express irritability, and easily lose patience, which can lead to expressions that are inappropriate for the social norms and not very suitable for the context [24]. Extrapolating the previous comments on working memory, emotional lability could also be considered a marker that is present in ADHD persistence.

There is evidence that ADHD is associated with lower self-esteem in adulthood [19, 20], but our data indicate that a more negative self-image is largely associated with the persistence of the disorder. The ADHD-R group did not report having more significant self-concept problems than the N-ADHD group, which could suggest that remission protects the individual from these types of difficulties. However, only the analysis of the participants’ data related to the self-concept evaluation in the initial assessment (12 years earlier) and in the current study could help to clarify this question. By contrast, on the two behavior problems most closely linked to ADHD, the remittent group showed more significant problems than the N-ADHD group. The persistence of attention problems has frequently been pointed out as a relevant difficulty that remains throughout the life cycle of people with this disorder, but the high scores on hyperactivity/restlessness are surprising in light of the commonly held belief that the activity level declines in adults with ADHD [37, 52].

Undoubtedly, the differences in the comparisons of the three groups, ADHD-P, ADHD-R, and N-ADHD, are fairly evident in different functional impairments [19–23]. In all the domains evaluated, that is, family, school, life skills, social activities, and risky activities, ADHD-P individuals experience significantly more impairment than the N-ADHD group. In addition, in aspects related to family life, academic activities, and risky activities (e.g., risky driving, drugs, alcohol), the ADHD-P group was significantly more affected than the ADHD-R group. These results would be expected, given the considerable severity of the ADHD-P group, reflected in the results on the four EF, inhibition, shift, working memory, and plan/organize, and on the ADHD associated behaviors evaluated.

In the first structural equation model used to investigate how EF predicted total functional impairments, the effect of inhibition was positive and statistically significant, whereas shift and working memory only marginally predicted impairments in all groups. Plan/organize showed a moderation effect of group, affecting total impairment more in the ADHD-P group than in the ADHD-R group; this effect was not statistically significant in the N-ADHD group. Therefore, as in other adult follow-up studies, EF ratings were good predictors of impairments in major life activities [17, 25]. However, the most relevant finding was the group-specific plan/organize deficit in the ADHD-P group, suggesting that these skills are important domains that should be targeted in treatment.

In the second model examining functional impairment with associated ADHD behaviors, hyperactivity/restlessness and self-concept had equal effects across the three groups, ADHD-P, ADHD-R, and N-ADHD. In addition, the effect of hyperactivity was not statistically significant, whereas the effect of self-concept was positive and statistically significant. More importantly, inattention and impulsivity/emotional lability showed group-specific effects on total functional impairment. On the one hand, total functional impairments in ADHD-P individuals were more influenced by attention problems than in the N-ADHD group, whereas this effect was not statistically significant in the ADHD-R group. On the other hand, the effects of impulsivity/emotional lability were much larger in the ADHD-R group than in the other two groups, which did not significantly differ in these effects.

Our study was uniquely suited to investigate the profile and functional impairments that characterize the persistence and remission of adult ADHD. The findings show that significant executive and behavioral impairments and, above all, adverse functional outcomes in family, school, life skills, social activities, and risky activities are related to the diagnostic persistence of ADHD. Recovery from the disorder is associated with better results on the EF, which are even more evident in the problems typically associated with ADHD. However, some deficits, such as plan/organize strategies, working memory and hyperactivity/restlessness, continue to be present in ADHD-R individuals, even though they no longer meet the criteria for the ADHD diagnosis. Thus, although the core ADHD symptoms may be in remission, other deficits that were not analyzed in sufficient depth in this study may negatively impact important outcomes (e.g., academic level, family environment, social support network, etc.)

Moreover, the results showed that some specific executive deficits and ADHD associated behavior problems contributed to the functional impairments in ADHD-R and ADHD-P. Thus, there was a higher group effect on plan/organize errors in the ADHD-P group than in the ADHD-R group, whereas the effect of the problems with impulsivity/emotional lability when examining functional impairments was greater in the ADHD-P group than in the ADHD-R group. In sum, the EF impairments in adult individuals with a diagnosis of ADHD in childhood are significant due to their presence in the persistence of the disorder, which is associated with functional impairments in domains of major life activities. Therefore, these processes are relevant from a practical point of view. Although they lack specificity and cannot strictly be considered diagnostic criteria for ADHD, behaviors reflecting executive problems in daily life activities must be taken into account by clinicians in the evaluation process to monitor adults who show weaknesses on these markers.

This study presents some main limitations related to the characteristics of the sample and the evaluation procedure. The small sample size increases the probability of Type II errors, so that some null findings could be related to a lack of statistical power. Moreover, the sample was composed only of cases diagnosed with ADHD-C in childhood that were followed up until the young adulthood stage. Most of the participants were males with ages from 18 to 24 years old. For these reasons, the findings cannot be generalized to other presentations of ADHD, older adults, or females. Furthermore, in the identification of the ADHD-P group, the presence of impairment was not taken into account. According to a recent systematic review, impairment should be considered a factor when rigorously determining the persistence of ADHD [1]. In addition, we relied on self-report questionnaires as the evaluation method. This procedure might have produced biases where the participants generalized the symptom severity and impairments across all the domains, regardless of the construct measured. In any case, self-reports have been shown to provide valid information, with high levels of agreement between clinician and patient ratings of ADHD symptoms and emotion dysregulation symptoms [53]. Likewise, the results might have been influenced by the overlapping of the contents on the BRIEF and the CAARS. However, the two measures seem to capture different aspects of adult ADHD: the BRIEF is more impairment-oriented, whereas the CAARS is more symptom-oriented [54]. Finally, it should be taken into account that the contribution of the variables included in this study was moderate, which suggests that other variables may also be at work. Factors in the family and extended social context, or even cognitive processes such as a sluggish cognitive, metacognitive, or motivational rhythm, could also contribute to explaining the results in terms of impairments in daily life.

## Conclusion

This study provides evidence that persistence of ADHD in young adulthood is associated with severe executive, behavioral, and functional impairments in family, social, academic, and risky activities. The remission of ADHD manifested more subtle problems that were linked specifically to attention and hyperactivity/impulsivity behaviors. Structural equation models indicated that global functional impairment was predicted in the ADHD-P group by plan/organize and attentional problems. Finally, the impulsivity/emotional lability was found to be a very important predictive factor of global functional impairment in the ADHD-R group.

In sum, the findings show that problems of adults with an ADHD childhood diagnosis do not usually disappear, and they encourage close collaboration between children’s and adults’ mental health services in performing the necessary follow-ups of this chronic disorder.

## Data Availability

Please contact Dr. Ana Miranda for data request.

## Abbreviations

ADHD: Attention deficit hyperactivity disorder
ADHD-P: ADHD persistent
ADHD-R: ADHD remittent
N-ADHD: Without ADHD
MANOVA: Multivariate Analyses of Variance
EF: Executive functioning
EL: Emotional lability
IMAGE: International Multicenter ADHD Genetics
IQ: Intellectual Quotient
DSM: Diagnostic and Statistical Manual of Mental Disorders
ADHD-C: ADHD combined
CAARS: Conners Adult Rating Scale ADHD-C
BRIEF: Behavior Rating Inventory of Executive Function
BRI: Behavior regulation index
MI: Metacognition Index
WFIRS-S: Weiss Functional Impairment Rating Scale Self-Report
CFI: Comparative fit index
SRMR: Square root mean residual
